# Case of Peritonitis—Attacks of Shivering—Inflammation of the Vena Portœ—Death

**Published:** 1846-07

**Authors:** 


*Case of Peritonitis—Attacks of Shivering—Inflammation of the vena portæ—death. Remarks on Inflammation of the portal vein in general.*—The patient was a man 26 years of age. When admitted into the hospital, he was complaining of a violent pain in the epigastrium; it shifted its locality a little in different positions of the body. Pyrexia, diarrhœa, great thirst, and a high-coloured state of the urine present. The case was regarded as one of peritonitis, and treated accordingly. On the third day, there was a well-marked shivering fit, followed by heat, but not by perspiration. Schoenlein says, that he suspected the commencement of some mischief in the portal system of veins. The rigors returned at irregular intervals; on some days there were several attacks. The treatment consisted in the inunction of mercurial ointment, the application of fomentations, and the internal use of calomel; the patient was suddenly and rather severely salivated. Dr. S. assures us that, in several cases of lesion of the portal system, he has observed decided benefit from the use of saline baths, (two pounds of common salt, and one ounce of muriate of lime being added to a usual bath;) and he accordingly ordered them for this patient. The icteric and febrile symptoms, however, continued to exist, and indeed gradually became worse. The patient died in the course of about two months.

*Dissection.*—On opening the abdomen, an abcess was found about midway between the umbilicus and the point of the sternum (the seat of the pain during life;) it was situated between the layers of the meso-colon. The abscess extended backwards in the direction of the vena portæ. This vessel was found to be considerably enlarged, and, when laid open, to be filled with pus. The matter extended into its ramifications, the inner membrane of which was observed to be decidedly thickened. The parenchymatous substance of the liver was not affected.

In his comment upon this case, Mr. Schoenlein directs the attention of the reader to that aphorism of Hippocrates, wherein it is said—"Those who suffer with violent fever rarely recover, if a shivering occurs on the sixth day." Our author regards the occurrence of this phenomenon, under such circumstances, to be a probable indication of the commencement of phlebitis somewhere. As to the *symptoms of inflammation of the portal vein*, one of the most constant is (as far as we can well judge from our hitherto limited experience,) a heavy oppressive pain about midway between the point of the ensiform cartilage and the umbilicus, and often, it may be, extending backwards in the direction of the spine; it is always increased by firm pressure. The usual signs of the biliary secretions being more or less imperfectly performed are generally present. Occasionally blood is passed with the stools: indeed, Dr. S. is of opinion that the disease of melœna is not unfrequently connected with the lesion which we are now describing. The accompanying fever is usually violent,—the *causus* of the ancient writers. Should the inflammation terminate in the formation of purulent matter, the fever will necessarily assume more of a typhoid, or perhaps of a hectic character. If, in place of pus, plastic lymph is effused into the cavity of the inflamed vessels, the superficial abdominal veins are apt to become very considerably dilated, and the spleen also to be so much distended that, in the course of a few days, it may be felt outwardly to be excessively enlarged. When this is the case, we may expect to have all the usual symptoms of spleen disease: viz. tendency to syncope, confusion of sight, hæmorrhage from the left nostril (singular precision)! and evacuation or vomiting, and, consequently, hæmorrhage from the bowels, which is almost always accompanied with extreme prostration. In one case, which has been described in Schmidt's Journal, (1840,) Schoenlein found, upon dissection, all the veins of the portal system quite obliterated.—*Med. Chir. Review, from Schoenlein's Clinical Lectures.*

